# Promising novel biomarkers and candidate small-molecule drugs for lung adenocarcinoma: Evidence from bioinformatics analysis of high-throughput data

**DOI:** 10.1515/med-2021-0375

**Published:** 2021-12-21

**Authors:** Chengrui Li, Yufeng Wan, Weijun Deng, Fan Fei, Linlin Wang, Fuwei Qi, Zhong Zheng

**Affiliations:** Department of Anesthesiology, Lianshui People’s Hospital Affiliated to Kangda College of Nanjing Medical University, Huai’an, People’s Republic of China; Department of Respiratory Medicine, The Affiliated Huai’an Hospital of Xuzhou Medical University and The Second People’s Hospital of Huai’an, Huai’an, Jiangsu 223002, People’s Republic of China; Department of Thoracic Surgery, Lianshui People’s Hospital Affiliated to Kangda College of Nanjing Medical University, Huai’an, People’s Republic of China; Department of Anesthesiology, The First People’s Hospital of Taicang City, Taicang Affiliated Hospital of Soochow University, Suzhou, People’s Republic of China; Department of Respiratory Medicine, The First People’s Hospital of Taicang City, Taicang Affiliated Hospital of Soochow University, Suzhou, People’s Republic of China

**Keywords:** lung adenocarcinoma, bioinformatics analysis, prognosis, candidate small molecules, novel biomarkers

## Abstract

Lung adenocarcinoma (LUAD) is the most common subtype of non-small cell lung cancer associated with an unstable prognosis. Thus, there is an urgent demand for the identification of novel diagnostic and prognostic biomarkers as well as targeted drugs for LUAD. The present study aimed to identify potential new biomarkers associated with the pathogenesis and prognosis of LUAD. Three microarray datasets (GSE10072, GSE31210, and GSE40791) from the Gene Expression Omnibus database were integrated to identify the differentially expressed genes (DEGs) in normal and LUAD samples using the limma package. Bioinformatics tools were used to perform functional and signaling pathway enrichment analyses for the DEGs. The expression and prognostic values of the hub genes were further evaluated by Gene Expression Profiling Interactive Analysis and real-time quantitative polymerase chain reaction. Furthermore, we mined the “Connectivity Map” (CMap) to explore candidate small molecules that can reverse the tumoral of LUAD based on the DEGs. A total of 505 DEGs were identified, which included 337 downregulated and 168 upregulated genes. The PPI network was established with 1,860 interactions and 373 nodes. The most significant pathway and functional enrichment associated with the genes were cell adhesion and extracellular matrix-receptor interaction, respectively. Seven DEGs with high connectivity degrees (ZWINT, RRM2, NDC80, KIF4A, CEP55, CENPU, and CENPF) that were significantly associated with worse survival were chosen as hub genes. Lastly, top 20 most important small molecules which reverses the LUAD gene expressions were identified. The findings contribute to revealing the molecular mechanisms of the initiation and progression of LUAD and provide new insights for integrating multiple biomarkers in clinical practice.

## 1 Introduction

**Figure 1:**
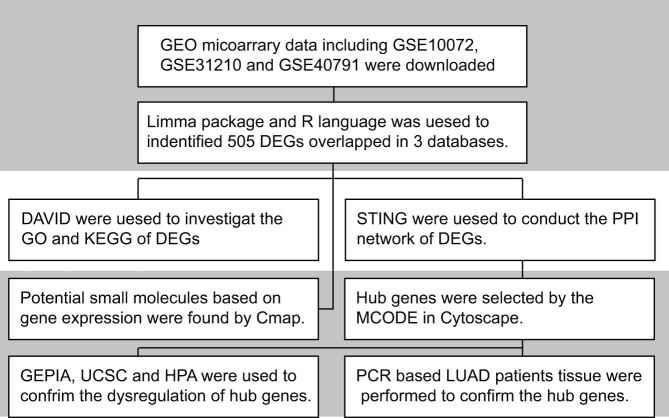
Study workflow for the identification of primary genes and pathways in LUAD.

Lung cancer is a serious and common disease that causes approximately 1.6 million deaths annually and ranks first in causing mortality and morbidity among all cancer types [1]. Malignant epithelial tumors is considered as most commonly diagnosed lung cancer that is further classified into non-small cell lung cancer (NSCLC) and small cell lung cancer (SCLC) [2]. NSCLC comprises nearly 85–90% of the cases, and lung adenocarcinoma (LUAD) is considered the most common subtype. Despite the improvements in multimodal treatments for LUAD, including chemotherapy, targeted therapy, surgery, and radiation therapy, the survival benefits are far from ideal [3–5]. Advanced metastatic disease is observed in nearly 50% of the patients with LUAD, and the overall 5-year survival rate in such patients is <4%. The occurrence and development of LUAD are not only related to mutation, deletion, and genomic amplification but also epigenetic modifications. The involvement of a large number of biomarkers in transcription as well as post-transcriptional regulations via phosphorylation and methylation is associated with the development of cancer [6,7]. Various intracellular signaling molecules play a significant role in tumor invasion and metastasis, leading to poor prognosis in patients with LUAD [8–11]. Therefore, the identification of novel biomarkers and understanding their molecular mechanisms will contribute to enhancing our knowledge regarding the initiation and progression of LUAD. Previous studies on gene expression have demonstrated that DNA alterations and mutations play vital roles in the malignant transformation of LUAD [12]. Recent studies on LUAD have focused on epithelial-mesenchymal transition, which is an important mechanism underlying the initiation of many metastatic diseases, including LUAD [13–15]. In addition, signaling molecules, especially liver kinase B1[16] and Kirsten rat sarcoma viral oncogene, are involved in the invasiveness and metastasis of LUAD [12]. The GSE10072, GSE31210, and GSE40791 gene expression profiles were integrated from the Gene Expression Omnibus (GEO) database to explore novel biomarkers associated with the pathogenesis and prognosis of LUAD and identify the differentially expressed genes (DEGs) in LUAD and adjacent normal tissue. Meanwhile, we mined the “Connectivity Map” (CMap) database to explore some candidate small molecules which have the ability to reverse the gene expression changes in LUAD. Based on this, seven novel biomarkers were identified that might contribute to understanding the molecular mechanisms associated with the occurrence and progression of LUAD. These biomarkers are significant in the diagnosis and prognosis of patients with LUAD. In summary, this study aimed to provide new insights into this multi-gene hereditary disease and/or the diagnosis, prognosis, and targeted treatments of LUAD based on the novel biomarkers. Figure 1 depicts our study workflow.

## 2 Materials and methods

### 2.1 Data resources

We downloaded and assessed the GSE10072, GSE31210, and GSE40791 gene expression profiles from the GEO database (https://www.ncbi.nlm.nih.gov/geo/) to investigate the DEGs in normal and LUAD samples. GEO is a public repository of high-throughput microarray works for documenting experimental data. The RNA profiles were subjected to GPL96 (GSE10072) (Affymetrix Human Genome U133A Array) and GPL570 (Affymetrix Human Genome U113 Plus 2.0 Array). In our study, a total of 169 normal samples and 378 LUAD samples were acquired, which included 49 normal samples and 58 LUAD samples in the GSE10072 profile, 20 normal and 226 LUAD samples in the GSE31210 profile, and 100 normal and 94 tumor samples in the GSE40791 profile.

### 2.2 Identification of DEGs

We downloaded the original CEL files and categorized them into two groups, namely normal and LUAD groups. The Bioconductor affy package (https://www.bioconductor.org/) was used to transform raw data into expression values and for data standardization [17]. The empirical Bayes method in the limma package was used for the significance analysis to identify DEGs in the normal and LUAD samples [18]. A *P* value <0.05 and |log FC| >1 were considered the cut-off criteria for selecting the significant DEGs.

### 2.3 Functional enrichment analysis

Gene Ontology (GO) enrichment analysis was performed with the Database for Annotation Visualization and Integrated Discovery (DAVID) tool to explore the overlapping DEG-associated cellular components, molecular functions, and biological processes. It is a commonly utilized online biological information database, which provides information regarding genes as well as comprehensive annotation information regarding the functions of proteins (version 6.7; https://david.ncifcrf.gov) [19–22]. Furthermore, we performed the KEGG pathway enrichment analysis to elucidate the potential signaling pathways associated with the overlapping DEGs. The KEGG database stores vast information about drugs, chemical substances, diseases, biological pathways, and genomes, and is commonly used to identify metabolic and functional pathways [23]. A *P* value of <0.05 was considered statistically significant.

### 2.4 Construction of the protein–protein interaction (PPI) network and module analysis

The Search Tool for the Retrieval of Interacting Genes (STRING, https://string-db.org/) database is an online tool designed to analyze the PPI data. The known DEGs were presented to the STRING database to explore their potential interactions. Cytoscape software was used to construct PPI networks based on a combined score of >0.4 [24]. Subsequently, from the PPI networks, significant modules were screened using the Molecular Complex Detection (MCODE) algorithm based on the following parameters: maximum depth = 100, *k*-core = 2, node score cutoff = 0.2, and degree cutoff = 10 [25]. In addition, pathway enrichment and functional analyses were performed on the most significant modules. BiNGO, a Cytoscape plugin from the Networks GO tool, was utilized to perform and visualize the biological process analysis of the module DEGs [26]. Further, the UCSC Cancer Genomics Browser (https://genome-cancer.ucsc.edu) was utilized to construct the hierarchical clustering of the module genes.

### 2.5 Analysis and validation of hub genes

The cBioPortal online platform (https://www.cbioportal.org) was used to establish the hub genes and their co-expression gene networks. Furthermore, an interactive web application tool, known as gene expression profiling interactive analysis (GEPIA), as well as the genotype-tissue expression and the cancer genome atlas databases were utilized to confirm the reliability of the expression levels of hub genes in LUAD and normal tissues. The GEPIA database contained 9,736 tumor samples and 8,587 normal samples [27–29]. In addition, we explored the prognostic value of hub genes on the GEPIA platform. The hazard ratio was computed based on 95% confidence intervals. Boxplot graphs and Kaplan–Meier curve were plotted to represent the data and illustrate the connections between patient prognosis and gene expression. The online tool, human protein atlas (HPA, www.proteinatlas.org) database, was utilized to determine the protein expression of hub genes in normal and LUAD tissues from the clinical samples. The antibody information can be accessed from https://www.proteinatlas.org/ENSG00000090889-KIF4A/antibody.

### 2.6 Real-time quantitative polymerase chain reaction (qPCR)

TRIzol reagent (Invitrogen, Carlsbad, CA) was utilized according to the manufacturer’s protocol to extract total RNA from non-tumorous and tumor tissues. For 0.5 h at 37°C, DNase I digestion (Fermentas, MD, USA) was subjected with 2 μg total RNA. The Omniscript Reverse Transcription kit (Qiagen, Valencia, CA) was used to synthesize the cDNA. The EvaGreen Master Mix (Biotium Inc., Hayward, CA) was used to perform the real-time qPCR assay. The qPCR amplification conditions were as follows: for 120 s at 95°C, following for 15 s at 95°C: 40 cycles, for 45 s annealing temperature. A triplicate was used to run each sample. With the help of the 2^−ΔΔCt^ relative quantification method, the *C*_t_ value of GAPDH (endogenous reference) was utilized to normalize the relative expression levels of each target gene. Primers are presented as:

Each reaction was carried out on the Eppendorf Mastercycler ep realplex system (2S; Eppendorf, Hamburg, Germany) based on the below cycling parameters, for 2 min at 95°C, following at 95°C for 15 s: 40 cycles, for 45 s at 60°C. The experimental protocol was established based on the ethical guidelines of the Declaration of Helsinki. The Human Ethics Committee at Taicang Hospital (No. 2018-K020) granted the ethical permissions. Written informed consent was obtained from the individuals or guardians of the participants.

## 3 Results

### 3.1 Identification of DEGs in LUAD

**Figure 2:**
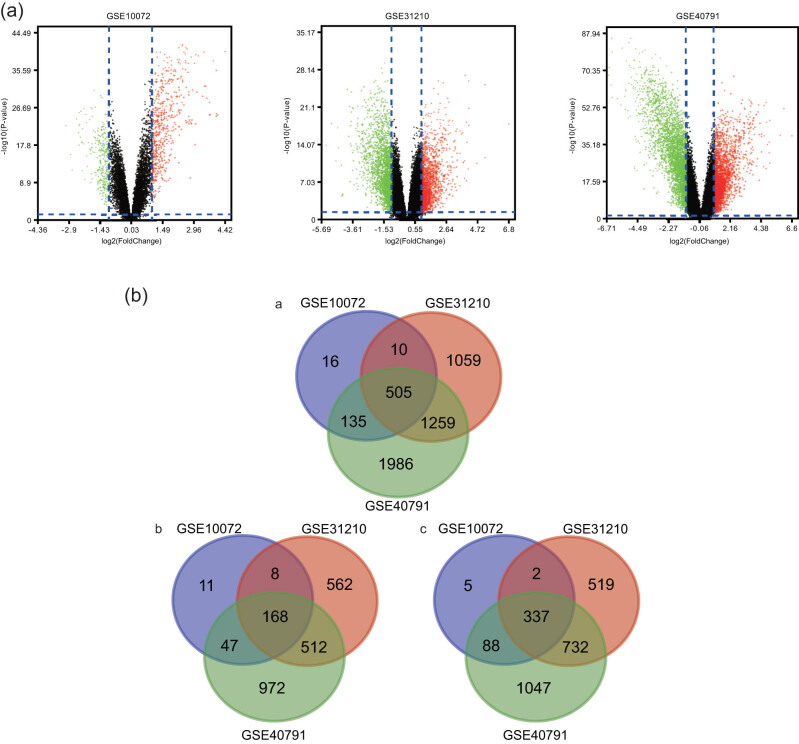
(a) Volcano plot of the gene expression profile data in LUAD and normal tissues in each dataset. Black dot: non-DEGs; green dot: substantially downregulated genes; red dot: substantially upregulated genes. |log 2 FC| >1 and *P* <0.05 were considered significant and (b) ((a) Venn diagram of the 505 overlapping DEGs from the GSE10072, GSE31210, and GSE40791 datasets. (b) Upregulated overlapping DEGs; and (c) Downregulated overlapping DEGs).

A total of 505 overlapping DEGs expressed in LUAD tissue identified using the limma package were extracted from the GSE10072, GSE31210, and GSE40791 datasets. Figure 2a presents the volcano plot of the gene expression profile in each dataset. Further, the overlapping DEGs have been presented in Venn diagrams (Figure 2b(a)), which included 337 downregulated genes (Figure 2b(c)) and 168 significantly upregulated genes (Figure 2b(b)).

### 3.2 Enrichment analyses

**Figure 3:**
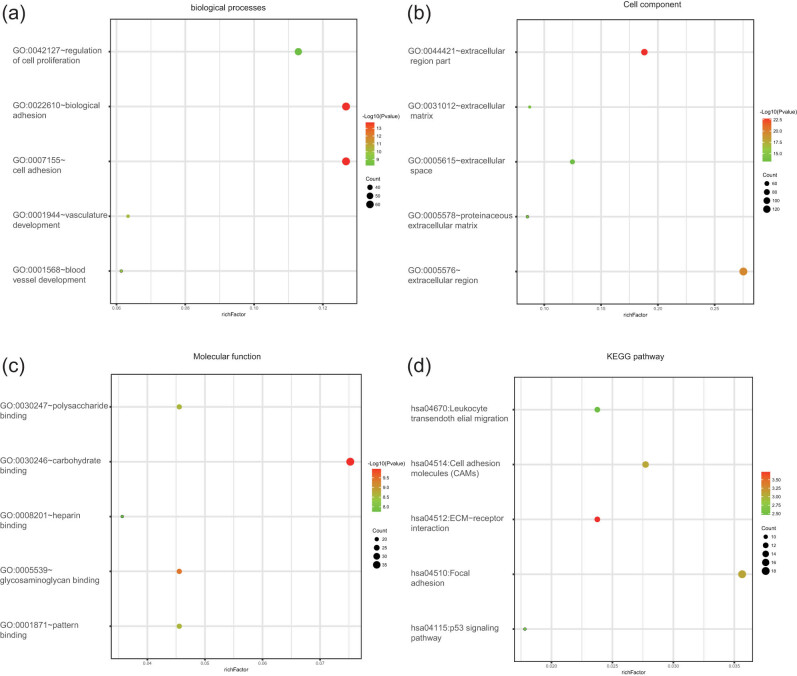
Functional and signaling pathway analyses of the overlapping DEGs in LUAD. (a) Biological process, (b) cellular component, (c) molecular function, and (d) KEGG pathways.

Pathway enrichment and functional analyses were performed on the upregulated and downregulated genes using the DAVID tool. For biological processes, significant enrichment was observed in blood vessel development, cell adhesion, biological adhesion, vasculature development, and cell proliferation regulation in the upregulated and downregulated DEGs. Cell component analysis revealed the involvement of these DEGs in the extracellular space, “extracellular matrix, proteinaceous extracellular matrix, extracellular region, and extracellular region part.” Likewise, the changes in molecular functions of the DEGs demonstrated significant enrichment in heparin, polysaccharide, pattern, glycosaminoglycan, and carbohydrate bindings. Additionally, KEGG pathway enrichment analysis revealed a close association among the DEGs and leukocyte transendothelial migration, p53 signaling pathway, cell adhesion molecules, focal adhesion, and ECM-receptor interactions (Table 1 and Figure 3).

**Table 1:** Functional and pathway enrichment analysis of the overlapping DEGs

Category	Term	Count	*P* value
GOTERM_BP_FAT	GO:0007155∼cell adhesion	64	2.02 × 10^−14^
GOTERM_BP_FAT	GO:0022610∼biological adhesion	64	2.12 × 10^−14^
GOTERM_BP_FAT	GO:0001944∼vasculature development	32	5.91 × 10^−11^
GOTERM_BP_FAT	GO:0001568∼blood vessel development	31	1.50 × 10^−10^
GOTERM_BP_FAT	GO:0042127∼regulation of cell proliferation	57	5.75 × 10^−9^
GOTERM_CC_FAT	GO:0044421∼extracellular region part	95	1.11 × 10^−23^
GOTERM_CC_FAT	GO:0005576∼extracellular region	139	2.88 × 10^−20^
GOTERM_CC_FAT	GO:0005578∼proteinaceous extracellular matrix	43	4.65 × 10^−15^
GOTERM_CC_FAT	GO:0031012∼extracellular matrix	44	1.39 × 10^−14^
GOTERM_CC_FAT	GO:0005615∼extracellular space	63	6.59 × 10^−14^
GOTERM_MF_FAT	GO:0030246∼carbohydrate binding	38	1.07 × 10^−10^
GOTERM_MF_FAT	GO:0005539∼glycosaminoglycan binding	23	3.83 × 10^−10^
GOTERM_MF_FAT	GO:0001871∼pattern binding	23	2.46 × 10^−9^
GOTERM_MF_FAT	GO:0030247∼polysaccharide binding	23	2.46 × 10^−9^
GOTERM_MF_FAT	GO:0008201∼heparin binding	18	1.83 × 10^−8^
KEGG_PATHWAY	hsa04512:ECM-receptor interaction	12	1.82 × 10^−4^
KEGG_PATHWAY	hsa04510:Focal adhesion	18	8.11 × 10^−4^
KEGG_PATHWAY	hsa04514:Cell adhesion molecules (CAMs)	14	8.37 × 10^−4^
KEGG_PATHWAY	hsa04115:p53 signaling pathway	9	0.00281
KEGG_PATHWAY	hsa04670:Leukocyte transendothelial migration	12	0.003238

### 3.3 Construction of the PPI network and module analysis

**Figure 4:**
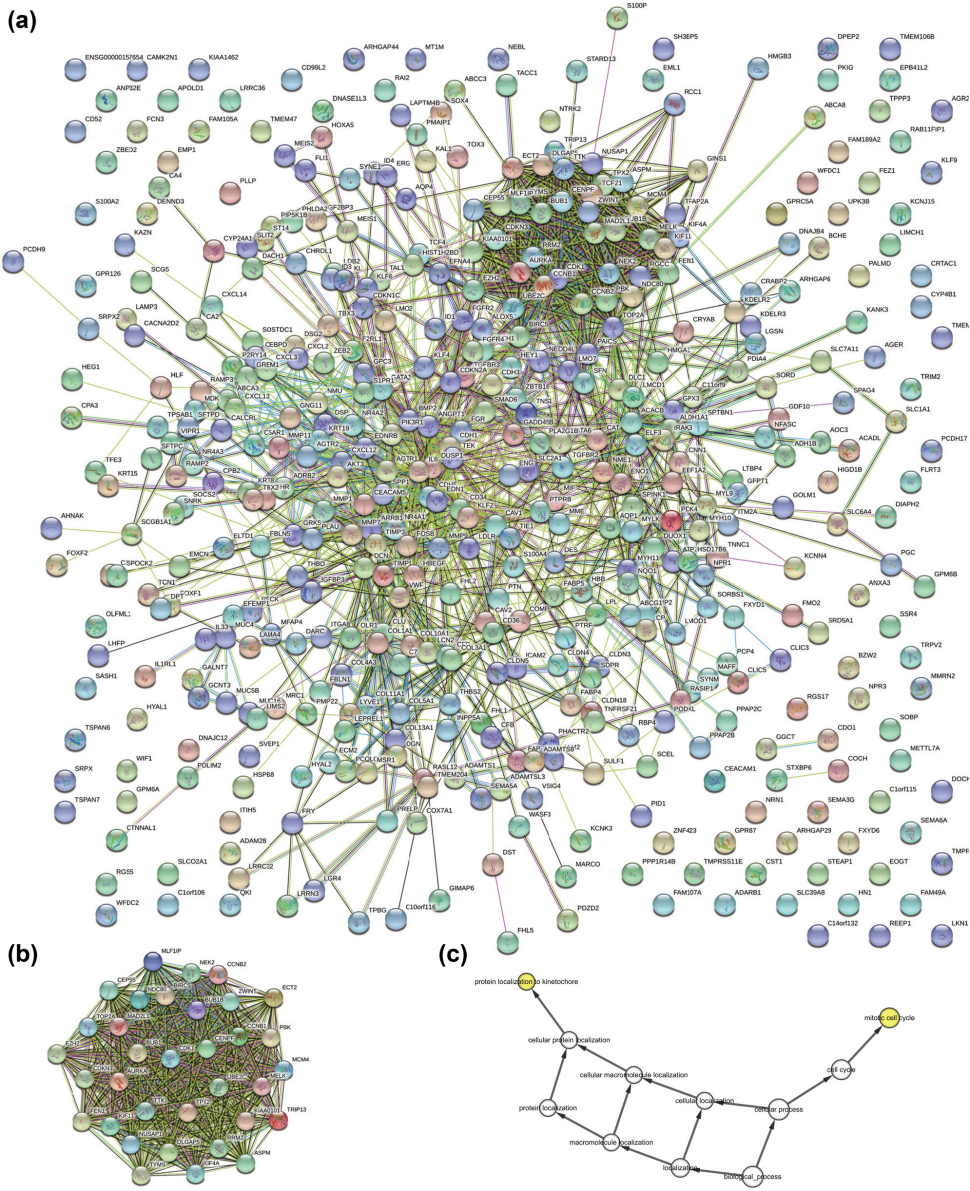
(a) PPI network construction and module analysis. (b) The most significant module. (c) The biological process analysis of the module genes by BiNGO. The color depth of the nodes represents the *P* value correction. The node size represents the total genes involved.

**Figure 5:**
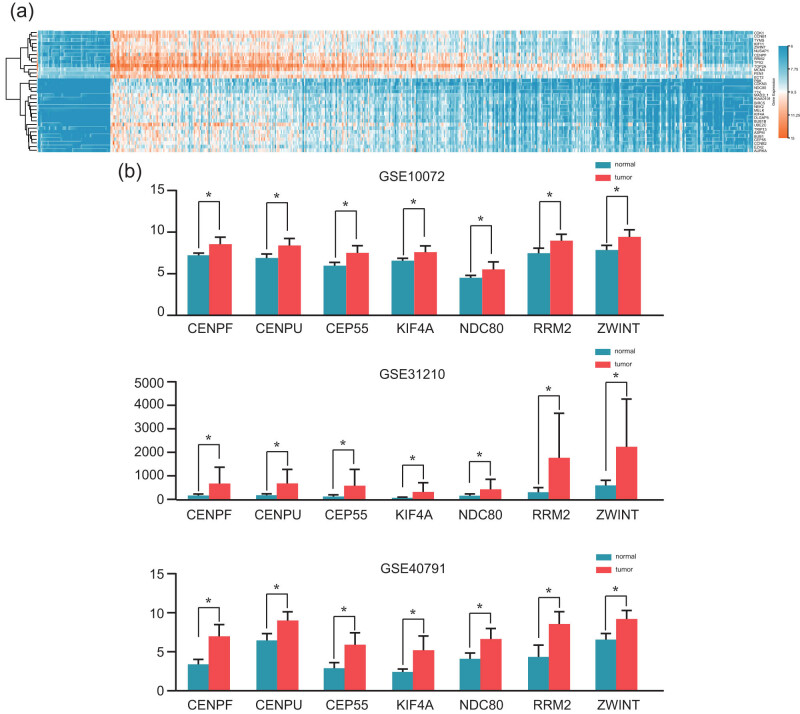
(a) Expression levels of hub genes in the normal and LUAD tissues in the three datasets. (b) The heatmap of the module genes in the normal and LUAD samples based on the UCSC database.

Using the Cytoscape software, the PPI network of the DEGs was established with 1,860 interactions and 373 nodes based on the STRING database (Figure 3a). Further, three high-scoring modules were extracted from the PPI network using the MCODE plugin (Figure 4a). The pathway enrichment analysis of the most significant modules is shown in Table 2. The module genes were significantly associated with the p53 signaling pathway, “progesterone-mediated oocyte maturation, oocyte meiosis, and cell cycle” and DNA replication pathways. Biological process analysis revealed a significant relationship between the module genes and the mitotic cell cycle, cell cycle, and cellular processes (Table 2). In addition, hierarchical clustering indicated that LUAD tissues could be differentiated from noncancerous tissues by the hub genes (Figure 5a). The ZWINT, RRM2, NDC80, KIF4A, CEP55, CENPU, and CENPF genes that demonstrated high connectivity degrees were considered the hub genes. The aforementioned hub genes were significantly upregulated in LUAD tissues in every dataset (Figure 5b). Table S1 presents the functional roles and complete names of the hub genes.

**Table 2:** Functional and pathway enrichment analysis of genes in the most significant modules

Term	ID	Input number	*P* value
Cell cycle	hsa04110	8	3.31 × 10^−13^
Oocyte meiosis	hsa04114	6	1.86 × 10^−9^
Progesterone-mediated oocyte maturation	hsa04914	5	3.72 × 10^−8^
p53 signaling pathway	hsa04115	4	5.87 × 10^−7^
DNA replication	hsa03030	2	0.000548

### 3.4 Analysis and validation of hub genes

**Figure 6:**
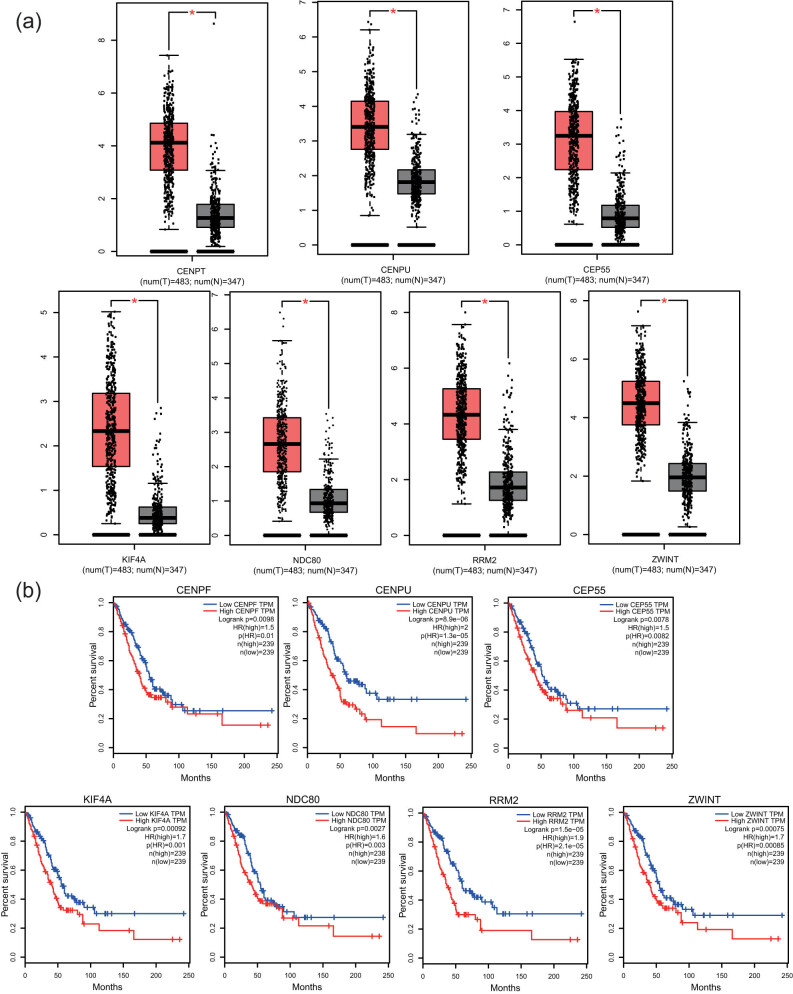
The (a) expression levels and (b) prognostic values of hub genes based on the GEPIA database.

**Figure 7:**
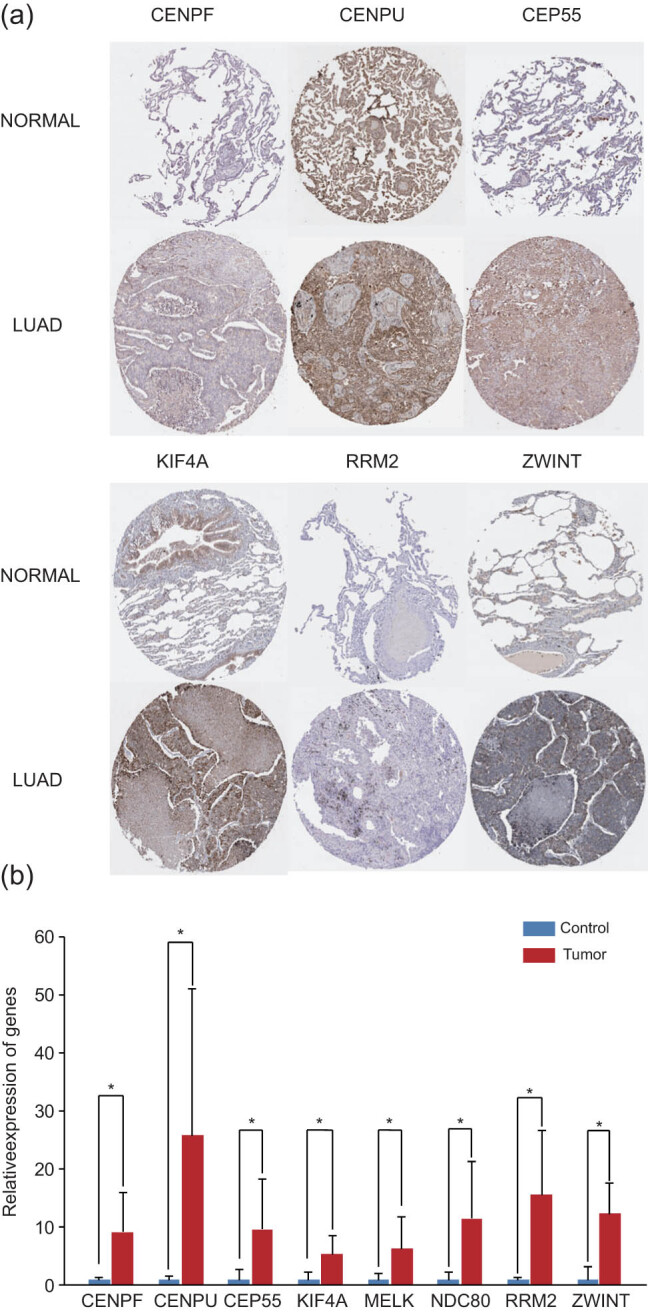
(a) Results of the representative immunohistochemistry staining showing the protein level expressions of the hub genes in normal and LUAD tissues. (b) List of 20 significant small-molecule drugs with the ability to reverse the tumoral status of LUAD.

**Figure 8:**
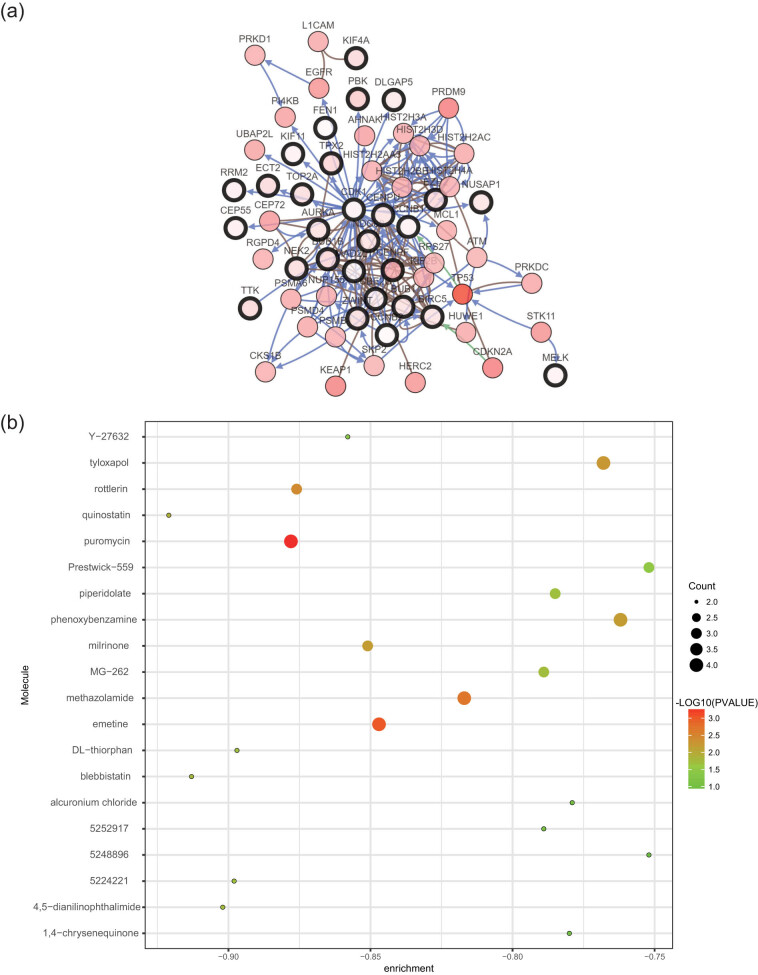
(a) Hub genes and their co-expression gene networks constructed using the cBioPortal tool. Hub genes and their co-expression genes are represented by nodes with thick and thin outlines, respectively. (b) Pop plot of the top 20 small molecules with the ability to reverse the gene expression in LUAD. (c) qPCR validation of hub genes in the seven paired LUAD samples. *P <0.05.

**Figure S1:**
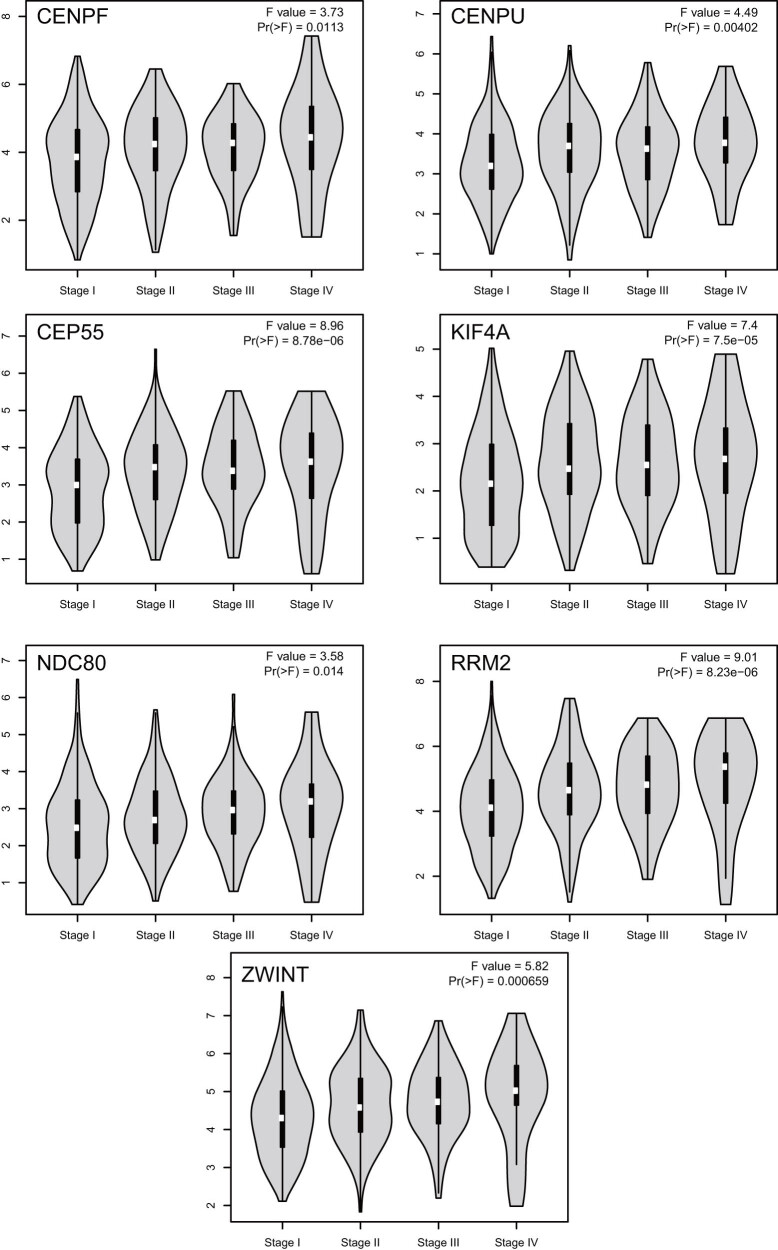
The relationship between the expression and stages of hub genes.

**Figure S2:**
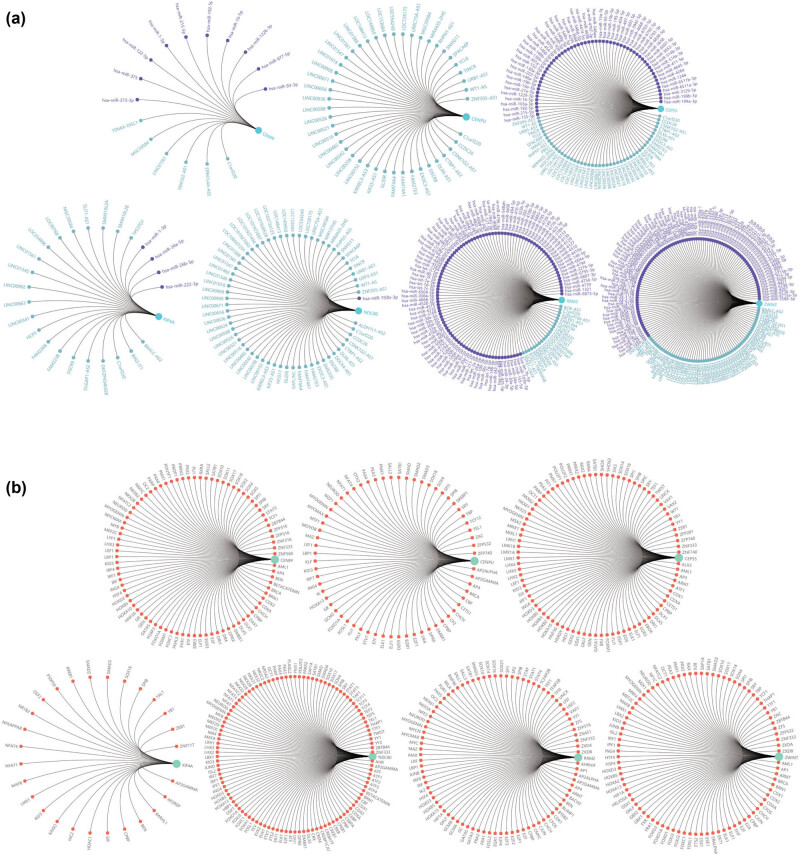
(a) The lncRNA-miRNA-mRNA regulatory network established with the GCBI platform. Blue nodes: targeted miRNA; purple nodes: related lncRNA. (b) The potential transcription factors that could be involved in regulating the expression of hub genes.

The genomics database was utilized to validate the correlation between the cBioPortal and HPA databases and the clinical characteristics of LUAD for cancer and hub gene expression. The GEPIA database mining revealed significant differences in the expressions of NUSAP1, KCNJ9, KCNJ3, HTR2A, GRIN1, GABRD, DLG2, and CHRM1 genes in normal and LUAD tissues (Figure 6a). The results confirmed a close correlation between the expression levels of hub genes and the onset of LUAD. The data of 478 patients with LUAD were obtained from the GEPIA database to analyze the overall survival that was then categorized as high and low expressions. A correlation was observed between the upregulation of the ZWINT, RRM2, NDC80, KIF4A, CEP55, CENPU, and CENPF genes and worse overall survival in patients with LUAD (Figure 6b). Simultaneously, for predicting the LUAD patients’ survival, the significant prognostic biomarkers could be represented by the ZWINT, RRM2, NDC80, KIF4A, CEP55, CENPU, and CENPF expression levels. Considering that the gene expression and associated protein content are not always consistent [30], the protein levels of the hub genes were further analyzed in clinical LUAD tissues. Furthermore, the immunohistochemical staining results based on the HPA database revealed a significantly high positivity of the ZWINT, RRM2, NDC80, KIF4A, CEP55, CENPU, and CENPF genes in cancer tissues compared to adjacent normal tissues (Figure 7). The cBioPortal online platform was utilized to construct the hub genes and their co-expression gene networks (Figure 8a). Potential transcription factors were predicted to further explore the molecular mechanism of the hub genes, which were involved in the regulation of their expression (Figure S1). In addition, the regulatory networks of mRNA, miRNA, and lncRNA were constructed with the help of the Gene-Cloud Biotechnology Information platform (Figure S2).

### 3.5 Identification of related active small molecules

Gene Set Enrichment Analysis was performed by uploading the upregulated and downregulated DEG groups in the CMap database to screen potential therapeutic drugs and identify candidate small molecules for LUAD. Subsequently, they were matched with the treatment of small molecules. This procedure aimed to identify small molecules with the ability to reverse the gene expression in LUAD. Figure 8b lists the 20 most significant small molecules along with their *P* values and enrichment scores. Blebbistatin (enrichment score, −0.913) and quinostatin (enrichment score, −0.921) were associated with high negative scores and were considered the most promising small molecules for reversing the gene expressions in LUAD. Such small-molecule drugs have the potential to cure LUAD and can provide new ideas leading to future developments for the treatment of LUAD. Although these potential small molecules may prove useful in further elucidating the etiology of LUAD, additional research is necessary to confirm their involvement in the condition.

### 3.6 Gene expression evaluation in LUAD

Seven pairs of tumor and adjacent non-tumorous tissues were selected to verify the expressions of the ZWINT, RRM2, NDC80, KIF4A, CEP55, CENPU, and CENPF genes. The qPCR assay was performed in LUAD and adjacent non-tumorous tissues to quantify the relative mRNA expressions of the ZWINT, RRM2, NDC80, KIF4A, CEP55, CENPU, and CENPF genes. The results revealed that the average mRNA expression levels of the aforementioned genes were considerably high in LUAD tissues compared to non-tumorous tissues (*P* <0.05, Figure 8c).

## 4 Discussion

In recent years, the development of high-throughput sequencing technologies has brought hope for understanding epigenetic changes and deciphering key genetic changes in the initiation and progression of tumors [31–33]. In prognostic analysis, integrated bioinformatics analysis plays a significant role in hub node discovery from the PPI network and screening of DEGs [34]. The particular technology has been extensively utilized for identifying potential novel biomarkers associated with the diagnosis, prognosis, and treatment of LUAD [35–37]. In this study, we identified the DEGs in LUAD and adjacent normal tissues by integrating three microarray datasets from the GEO database. By this, we aimed to identify novel prognostic and diagnostic biomarkers and promising therapeutic targets for LUAD. Furthermore, we identified small-molecule drugs for the treatment of LUAD and pharmaceuticals that can block tumor formation. These findings might provide new avenues and pharmacological mechanisms for novel therapeutic strategies for LUAD.

A total of 505 overlapping DEGs were identified, which included 337 downregulated and 168 significantly upregulated genes. The DEGs overlapping with the incidence and development of LUAD have the potential to be strongly linked with the condition and may be considered potential biomarkers for the diagnosis, prognosis, and treatment. We performed the GO enrichment analysis on the overlapping DEGs to identify the possible pathways associated with the pathogenesis of LUAD. Among the biological processes, the three significant functions were vasculature development, biological adhesion, and cell adhesion. For the DEGs, enriched molecular functions were primarily observed in pattern, glycosaminoglycan, and carbohydrate bindings. The changes in cell components were primarily related to the proteinaceous extracellular matrix, extracellular region, and extracellular region part. In addition, five KEGG pathways were enriched in the overlapping DEGs, namely cell adhesion molecules, focal adhesion, ECM-receptor interaction, and the p53 signaling pathway. This mechanism involving engagement of the ECM receptor occurs during the growth and invasion of several cancers. A study reported that Twist2 regulates the expressions of CD44 and ITGA6 in the ECM-receptor interaction pathways, thereby stimulating the invasion and proliferation of kidney cancer cells. Poor infiltration of CD^8+^ T lymphocytes is associated with higher FAK expression, and FAK activity has been shown to be associated with tissue fibrosis. Inhibiting FAK is effective in limiting tumor growth and enabling patients to live longer [38, 39]. Many biological activities, including energy metabolism, are regulated by the tumor suppressor gene, p53. Cancer may be associated with metabolic abnormalities, which are thought to be caused by the deactivation of p53 function [40]. The following theories are in agreement with our assessment of the human genome. The PPI network of the overlapping DEGs revealed three distinct network components. The ZWINT, RRM2, NDC80, KIF4A, CEP55, CENPU, and CENPF genes with high connectivity degrees that were considerably upregulated in LUAD samples were chosen as hub genes. The GEPIA database was used to validate the results of the bioinformatics analysis and determine the expression levels and prognostic values of the genes in patients with LUAD. An even stronger trend in the expressions of hub genes was observed in the GEPIA database than in the GEO database, and this expression significantly affected the long-term outcomes of patients with LUAD. The overall longevity of patients with LUAD was negatively associated with higher levels of the ZWINT, RRM2, NDC80, KIF4A, CEP55, CENPU, and CENPF genes. These findings were validated in the HPA database, which revealed the expression and protein content of the hub genes. The qPCR assay was performed to evaluate the expressions of the hub genes in seven paired LUAD tissues. A similar gene expression tendency, as explained previously, was demonstrated in tumor and adjacent non-tumorous tissues by qPCR, thereby verifying the accuracy of our findings. The present study revealed the diagnostic, prognostic, and therapeutic values of the seven hub genes that might help in providing new insights on LUAD. To the best of our knowledge, no study has reported the role of these seven mRNAs in the initiation and progression of LUAD. We developed a molecular regulatory network of lncRNA–miRNA–mRNA for these genes and predicted the possible transcription factors associated with their regulation. The purpose was to explore the possibility of LUAD pathology being mediated by these mRNA molecules and build on our knowledge regarding such hereditary diseases. The construction of such regulatory networks will contribute to our understanding of the relationship between key genes and the initiation and progression of LUAD. Dysregulation of the miRNAs-COUP-TFII-FOXM1-CENPF axis plays a significant role in the metastasis of prostate cancer. CENPF, a master regulator of metastasis in prostate cancer, is inhibited by microRNA miR-101 and miR-27a [41]. CENPU is a novel transcriptional repressor that is related to various cancers. Downregulation of CENPU can suppress the proliferation of breast cancer cells and cycle progression and further increase apoptosis [42]. CEP55 promotes the proliferation and invasion of osteosarcoma cells through the AKT signaling pathway. KIF4A plays a vital role in cell division and kinesin-related proteins’ kinesin 4 subfamily’ member. The dys-regulation of KIF4A is associated with the outcome of several cancer types, including lung, cervical, oral, and breast cancer [43]. Chromosomal instability can be considered a common feature of cancer cells and may be involved in the initiation of tumors. NDC80 participates in maintaining chromosome stability. Therefore, abnormal expression of NDC80 may be closely related to tumorigenesis. Similarly, the expression levels of RRM2 and ZWINT have been reported to be associated with tumor formation [44]. However, to the best of our knowledge, no study has reported the potential mechanisms of the initiation and progression of LUAD. It has been shown by above studies that the seven mRNAs might also play a significant role in the occurrence and development of LUAD.

Based on the CMap database and overlapping genes, a potential small-molecule drug set was identified, which could reverse the status of LUAD. The candidate small molecules with highly significant negative enrichment values might reverse the LUAD-induced abnormal gene expressions. Analysis of such molecules will contribute to the development of novel, targeted therapeutic drugs for LUAD. The safety and efficacy of quinostatin (enrichment score, −0.921), the most significant small molecule associated with the LUAD cell status, have not been evaluated in any cancer, including LUAD. However, it is unclear how this will affect the relation between blebbistatin (a positive enrichment score of −0.913) and LUAD. Given the ability to fully reverse the gene expression, further research should be conducted on the usefulness of the aforementioned small molecules in LUAD. This will help to understand better the therapeutic mechanisms of such candidate small-molecule drugs in LUAD, as a result of the contribution of different gene expression profiles from patients with LUAD.

We identified seven genes that might explain the molecular mechanism of the onset and development of LUAD based on gene expression pattern mining and detailed bioinformatics analysis. LUAD can be diagnosed, monitored, and treated based on these potential new biomarkers. Furthermore, we performed several comprehensive analyses to establish their critical involvement in the pathophysiology of LUAD. Additionally, we identified a collection of therapeutic candidate small-molecule drugs, which will enable us to identify novel targeted treatments for LUAD in the future. Many unique biomarkers have been identified in LUAD, and our research provides a fresh understanding of the malignancy.

## Appendix

**Tabel S1:** The full name and functional roles of eight hub genes

Gene symbol	Full name	Function
CENPF	Centromere protein F	This gene encodes a protein that associates with the centromere-kinetochore complex. The protein is a component of the nuclear matrix during the G2 phase of interphase. In late G2 the protein associates with the kinetochore and maintains this association through early anaphase. It localizes to the spindle midzone and the intracellular bridge in late anaphase and telophase, respectively, and is thought to be subsequently degraded. The localization of this protein suggests that it may play a role in chromosome segregation during mitotis. It is thought to form either a homodimer or heterodimer. Autoantibodies against this protein have been found in patients with cancer or graft versus host disease
CENPU	Centromere protein U	The centromere is a specialized chromatin domain, present throughout the cell cycle, that acts as a platform on which the transient assembly of the kinetochore occurs during mitosis. All active centromeres are characterized by the presence of long arrays of nucleosomes in which CENPA (MIM 117139) replaces histone H3 (see MIM 601128). MLF1IP, or CENPU, is an additional factor required for centromere assembly
CEP55	Centrosomal protein 55	CEP55 (Centrosomal Protein 55) is a Protein Coding gene. Diseases associated with CEP55 include Multinucleated Neurons, Anhydramnios, Renal Dysplasia, Cerebellar Hypoplasia, And Hydranencephaly and Meckel Syndrome, Type 1. Among its related pathways are Cytoskeletal Signaling and DNA Damage.
KIF4A	Kinesin family member 4A	This gene encodes a member of the kinesin 4 subfamily of kinesin related proteins. The encoded protein is an ATP dependent microtubule-based motor protein that is involved in the intracellular transport of membranous organelles. This protein also associates with condensed chromosome arms and may be involved in maintaining chromosome integrity during mitosis. This protein may also be involved in the organization of the central spindle prior to cytokinesis. A pseudogene of this gene is found on chromosome X.
NDC80	NDC80, kinetochore complex component	This gene encodes a component of the NDC80 kinetochore complex. The encoded protein consists of an N-terminal microtubule binding domain and a C-terminal coiled-coiled domain that interacts with other components of the complex. This protein functions to organize and stabilize microtubule-kinetochore interactions and is required for proper chromosome segregation
RRM2	Ribonucleotide Reductase Regulatory Subunit M2	This gene encodes one of two non-identical subunits for ribonucleotide reductase. This reductase catalyzes the formation of deoxyribonucleotides from ribonucleotides. Synthesis of the encoded protein (M2) is regulated in a cell-cycle dependent fashion. Transcription from this gene can initiate from alternative promoters, which results in two isoforms that differ in the lengths of their N-termini. Related pseudogenes have been identified on chromosomes 1 and X.
ZWINT	ZW10 interacting kinetochore protein	This gene encodes a protein that is clearly involved in kinetochore function although an exact role is not known. It interacts with ZW10, another kinetochore protein, possibly regulating the association between ZW10 and kinetochores. The encoded protein localizes to prophase kinetochores before ZW10 does and it remains detectable on the kinetochore until late anaphase. It has a uniform distribution in the cytoplasm of interphase cells. Alternatively spliced transcript variants encoding different isoforms have been found for this gene
